# A *Leishmania*-specific hypothetical protein expressed in both promastigote and amastigote stages of *Leishmania infantum* employed for the serodiagnosis of, and as a vaccine candidate against, visceral leishmaniasis

**DOI:** 10.1186/s13071-015-0964-5

**Published:** 2015-07-11

**Authors:** Vivian T. Martins, Mariana C. Duarte, Miguel A. Chávez-Fumagalli, Daniel Menezes-Souza, Cecília S. P. Coelho, Danielle F. de Magalhães-Soares, Ana Paula Fernandes, Manuel Soto, Carlos A. P. Tavares, Eduardo A. F. Coelho

**Affiliations:** Departamento de Bioquímica e Imunologia, Instituto de Ciências Biológicas, Universidade Federal de Minas Gerais, Belo Horizonte, Minas Gerais Brazil; Programa de Pós-Graduação em Ciências da Saúde: Infectologia e Medicina Tropical, Faculdade de Medicina, Universidade Federal de Minas Gerais, Belo Horizonte, Minas Gerais Brazil; Departamento de Patologia Clínica, COLTEC, Universidade Federal de Minas Gerais, Belo Horizonte, Minas Gerais Brazil; Departamento de Parasitologia, Instituto de Ciências Biológicas, Universidade Federal de Minas Gerais, Belo Horizonte, Minas Gerais Brazil; RW Bioprospection Ltda, Belo Horizonte, Minas Gerais Brazil; Departamento de Medicina Veterinária Preventiva, Escola de Veterinária, Universidade Federal de Minas Gerais, Belo Horizonte, Minas Gerais Brazil; Departamento de Análises Clínicas e Toxicológicas, Faculdade de Farmácia, Universidade Federal de Minas Gerais, Belo Horizonte, Minas Gerais Brazil; Centro de Biología Molecular Severo Ochoa, CSIC-UAM, Departamento de Biología Molecular, Universidad Autónoma de Madrid, Madrid, Spain; Laboratório de Biotecnologia Aplicada ao Estudo das Leishmanioses, Departamento de Patologia Clínica, COLTEC, Universidade Federal de Minas Gerais, Avenida Antônio Carlos 6627, 31.270-901 Belo Horizonte, Minas Gerais Brazil

**Keywords:** *Leishmania spp*, Hypothetical proteins, BALB/c mice, Vaccine, Serodiagnosis, Canine visceral leishmaniasis

## Abstract

**Background:**

LiHyV is an antigenic hypothetical protein present in both promastigote and amastigote stages of *Leishmania infantum,* which was recently identified by an immunoproteomic approach. A recombinant version of this protein (rLiHyV) was evaluated as a diagnostic marker for canine VL (CVL). In addition, the prophylactic efficacy of the rLiHyV protein, and two of its CD8^+^ T cell epitopes, has been analyzed in a murine model of visceral leishmaniasis (VL).

**Methods:**

Initially, the rLiHyV protein was evaluated by an ELISA technique for the serodiagnosis of CVL. Secondly, vaccines composed of the recombinant protein and both chemically synthesized peptides, combined with saponin as an adjuvant; were administered subcutaneously into BALB/c mice. The cellular and humoral responses generated by vaccination were evaluated. In addition, the parasite burden and immune response were studied 10 weeks after *L. infantum* infection.

**Results:**

The rLiHyV protein was recognized by antibodies of VL dogs. No cross-reactivity was obtained with sera from dogs vaccinated with a Brazilian commercial vaccine, with sera from animals infected with *Trypanosoma cruzi*, *Babesia canis* and *Ehrlichia canis*, or those from non-infected animals living in an endemic area for leishmaniasis. After challenge with *L. infantum*, spleen cells of BALB/c mice vaccinated with rLiHyV/saponin stimulated with parasite antigens showed a higher production of IFN-γ, IL-12 and GM-CSF, than the same cells obtained from mice vaccinated with the individual peptides, or mice from control (inoculated with saline or saponin) groups. This Th1-type cellular response observed in rLiHyV/saponin vaccinated mice was accompanied by the induction of parasite-specific IgG2a isotype antibodies. Animals immunized with rLiHyV/saponin showed significant reductions in the parasite burden in the liver, spleen, bone marrow and in the lymph nodes draining the paws relative to control mice.

**Conclusions:**

The present study showed for the first time that the *L. infantum* LiHyV protein could be considered as a vaccine candidate against *L. infantum* infection, as well as a diagnostic marker for CVL.

## Background

Leishmaniasis comprises a spectrum of diseases caused by protozoan parasites of the genus *Leishmania*, which presents a high morbidity and mortality in the world [1]. About 350 million people are at risk of contracting the infection in 98 countries, where 1.0 to 1.5 million new cases of cutaneous leishmaniasis (CL), and 200,000 to 500,000 new cases of visceral leishmaniasis (VL) are registered annually [2]. The treatment of disease is based on the parenteral administration of pentavalent antimonials, however, increased parasite resistance and side effects observed in the patients have been important problems [3, 4]. Other drugs, such as amphotericin B and its liposomal formulations, pentamidine and miltefosine are encouraging, however, their toxicity and/or high cost had limited their use [5, 6].

Canine visceral leishmaniasis (CVL) due to *Leishmania infantum* is a major global zoonosis, which is endemic in approximately 70 countries worldwide [7, 8]. It cannot only be considered as a veterinary disease, since infected dogs are the main domestic reservoirs of parasites for human disease [7]. In this context, and aiming to reduce the transmission of parasites between dogs and humans, one could suggest that it is necessary to diagnose CVL as early as possible. The Brazilian Public Health authorities determine that, to a precise immunological diagnosis of CVL, serological tests, such as IFAT and ELISA, should be employed for the effective serodiagnosis of disease [9]. However, ELISA is hampered by some factors, such as by variable specificity when different antigenic preparations are employed, which can lead to the occurrence of false-positive results caused by related pathogens, such as *Trypanosoma cruzi* [10, 11], *Ehrlichia canis* and *Babesia canis* [12]; or in vaccinated dogs [13]. In addition, its sensitivity presents problems, mainly related to the fact that a high percentage of infected dogs present low antileishmanial serology, and they are classified as false-negative in the serological assays performed; depending on the nature of the antigen employed in the assay [13]. Therefore, the development of new strategies to diagnose leishmaniasis has become a priority.

Studies to evaluate vaccine candidates against leishmaniasis have demonstrated the need to develop a Th1 cell-mediated immune response, which is based on the production of cytokines, such as IFN-γ, IL-12, and others [14–19]. The induction of CD4^+^ Th1 cells specific to parasite proteins is crucial in controlling the infection caused by *Leishmania spp*. Cytokines such as IFN-γ are able to induce the production of nitric oxide and other compounds by infected phagocytic cells, thereby assisting in the control of parasites’ multiplication [20, 21]. By opposite, cytokines such as TGF-β, IL-4, IL-10, and IL-13 represent important disease promoting substances, leading to the suppression of Th1 response [22, 23]. Concomitantly to the role of CD4^+^ T cells, the cytotoxic activity performed by CD8^+^ T cells also contributes to protection against VL. These cells has been shown to protect against re-infection, as well as having a significant role in controlling the primary infection against *Leishmania spp.,* by increasing the Th1 immune response [19, 24–26].

Protozoan parasites of the genus *Leishmania* have a dimorphic asexual life cycle consisting of extracellular promastigotes, which multiply and develop within the alimentary tract of the sand fly vector, and intracellular amastigotes that multiply within the phagolysosomes of the macrophages of the mammal hosts [27]. In addition, most of the studies on *Leishmania spp.* vaccines have focused in the use of promastigote proteins [28]. Amastigote antigens have been far less evaluated as vaccine candidates against disease. However, these forms seem to be the more appropriate targets for the immune responses elicited by a vaccine, since after a few hours of initial infection and during the active disease, only the amastigote stage is present in the infected host tissues. In addition, in opposition to promastigotes, amastigotes reside inside host cells, and can be targets for CD8^+^ T cells [29, 30].

In the present study, the antigenic and immunoprophylactic properties of a recently described parasite protein have been studied. The *Leishmania-*specific hypothetical protein, LiHyV (NCBI accession number: XP_888524.1), was described as an antigen present in both promastigote and amastigote stages of *L. infantum* by an immunoproteomic approach [31]. A recombinant version of the LiHyV protein (rLiHyV) was evaluated as a diagnostic marker for CVL. In addition, the rLiHyV protein and two peptides containing two of its CD8^+^ T cells predicted epitopes were evaluated as vaccine candidates in a murine model of VL.

## Methods

### Ethics statement

All technical protocols using mice and dogs’ sera in the present study were performed in compliance with the National Guidelines of the Institutional Animal Care and Committee on the Ethical Handling of Research Animals (CEUA) from the Federal University of Minas Gerais (UFMG), which approved this study with the code number 043/2011.

### Mice and parasites

Female BALB/c mice (8 weeks age) were obtained from the breeding facilities of the Department of Biochemistry and Immunology, Institute of Biological Sciences (ICB), Federal University of Minas Gerais (UFMG); and were maintained under specific pathogen-free conditions. *Leishmania infantum* (MHOM/BR/1970/BH46) strain was used. Parasites were grown at 24 °C in Schneider’s medium (Sigma, St. Louis, MO, USA), which was supplemented by 20 % heat-inactivated fetal bovine serum (FBS, Sigma-Aldrich, USA), 20 mM L-glutamine, 200 U/mL penicillin, and 100 μg/mL streptomycin, at pH 7.4. The soluble *L. infantum* antigenic extract (SLA) was prepared from stationary-phase promastigotes of parasites, as previously described [32].

### Canine sera

The sample size of dogs’ sera consisted of 73 domestic animals (*Canis familiaris*), made up of males and females of different breeds and ages. Sera of CVL were selected on the basis of two *Leishmania spp.* serological tests (IFAT [IFAT- LVC Bio-Manguinhos kit] and ELISA [EIE-LVC Bio-Manguinhos kit], both from Biomanguinhos, Fiocruz, Brazil). Dogs with an IFAT titer < 1/40 or ELISA reactivity below the cut-off value indicated by the manufacturer were considered to be serologically seronegative. Animals with an IFAT titer > 1/40 and an ELISA value over the cut-off were considered to be serologically seropositive. Thus, symptomatic VL dogs (*n* = 16) were those positive by IFAT and ELISA, and presenting three or more of the following clinical symptoms: weight loss, alopecia, adenopathy, onychogryposis, hepatomegaly, conjunctivitis and exfoliative dermatitis on the nose, tail, and ear tips. In addition, all dogs presented positive parasitological results (PCR detection of *L. infantum* DNA). Non-infected dogs (*n* = 20) were selected from an endemic area of leishmaniasis (Belo Horizonte, Minas Gerais, Brazil), but presenting negative serological and parasitological results, as well as were free of any clinical suspicion or signals of leishmaniasis. Also, sera samples of non-infected animals immunized with a Brazilian commercial vaccine (Leish-Tec®) (*n* = 10), which were isolated in kennels to prevent their contact with transmitting vectors of leishmaniasis, were used. In addition, sera of dogs experimentally infected with *T. cruzi* (*n* = 13), *Ehrlichia canis* (*n* = 7) or *Babesia canis* (*n* = 7) were also used in the ELISA assays, in order to evaluate the cross-reactivity. These sera samples were obtained from previous projects, which evaluated the immune response in the infected animals [15, 33, 34]. Serum samples were provided by Prof. Maria Norma Mello (Department of Parasitology, UFMG, Belo Horizonte, Brazil), and Prof. Ana Paula Fernandes (Department of Clinical and Toxicological Analysis, UFMG, Belo Horizonte, Brazil).

### Cloning of DNA sequence coding for *L. infantum* hypothetical protein, LiHyV

The LiHyV (XP_888524.1) nucleotide and amino acid sequences were obtained from the National Center for Biotechnology Information (http//www.ncbi.nlm.nih.gov). The local alignment of the protein sequence against the available complete genomes of other organisms was performed by BLAST, which verified a high homology of amino acid sequence (>85 %) of this protein among the *L. major*, *L. amazonensis* and *L. infantum* species. The recombinant protein was obtained after cloning a *L. infantum* (MHOM/BR/1970/BH46) DNA fragment containing the protein-coding region. Initially, genomic DNA was extracted using a phenol:chloroform extraction, and this was used as a template. The *forward* (5′-GACGGATCCATGTCGGACGCATCATTC-3′) and *reverse* (5′-GCACAAGCTTAAGGGCGTAGAAAGCGGC-3′) primers were designed according to the DNA sequence of the \ORF described in the *L. infantum* genome sequence database (LinJ. 04.0160). The PCR product was cloned into the pGEM-T *easy* vector confirmed by sequencing and transferred to the pET21a expression vector (Novagen), using the *Bam*HI and *Hin*dIII restriction enzymes included in the primers for this purpose (underlined). Recombinant plasmid was transformed into *Escherichia coli* BL21 (DE3). The recombinant protein expression was performed by adding 1.0 mM IPTG (isopropyl-β-D-thiogalactopyranoside, Promega, Montreal, Canada), for 3 h at 37 °C, when cells were lysed by a homogenizer after five passages through the apparatus. The product was centrifuged at 13,000 × *g* for 20 min at 4 °C, while the rLiHyV protein, containing a tag of 6x residues of histidine, was purified under non-denaturing conditions, using a 5 mL HIS-Trap column (GE Healthcare Life Science) attached to an FPLC (GE Healthcare Life Science) system. After purification, the recombinant protein content was passed through a polymyxin-agarose column (Sigma), in order to remove residual endotoxins. The recombinant protein A2 was cloned and purified as previously described [32].

### *In silico* prediction of T-cell epitopes

The linear CD8^+^ T cells epitopes of LiHyV protein were predicted using BIMAS software (www-bimas.cit.nih.gov/molbio/hla_bind/), where peptides that presented a score higher than 100.0 were selected to be evaluated as vaccine candidates against VL. Two amino acid sequences were synthesized to be employed in the vaccination experiments: AGQSVPNSL (Peptide1) and VGIKNTAALFVLNATAI (Peptide2). The second peptide was originally composed of two different peptides (VGIKNTAAL and LFVLNATAI), which presented the best results of *in silico* prediction of CD8^+^ T cell epitopes in the analysis, besides a sequential position in the primary sequence of amino acids of the LiHyV protein. So, the peptides were commercially synthesized (Genscript®, USA)”.

### ELISA for the serodiagnosis of CVL

Previous titration curves were performed to determine the most appropriate antigen concentration and antibody dilution to be used. The recombinant LiHyV and A2 proteins, as well as SLA *L. infantum*-specific IgG antibodies were evaluated. For this, microtiter immunoassay plates (Falcon) were coated with rLiHyV, rA2 or SLA (1.0, 0.5 and 1.0 μg per well, respectively), diluted in 100 μL of coating buffer (50 mM carbonate buffer pH 9.6), for 18 h at 4 °C. The experimental protocol used in the ELISA assays was developed as previously described [21]. In addition, all sera samples were evaluated in the same day using the same reagents (lots, dilutions, etc.), aiming to reduce or eliminate the possible experimental variations and the interference in the interpretation of data that could occur if the ELISA assays had been performed on different days.

### Immunization and challenge infection

Mice (*n* = 8, per group) were vaccinated subcutaneously in their left hind footpad with 25 μg of rLiHyV, Peptide1 or Peptide2, all associated with 25 μg of saponin (*Quillaja saponaria* bark saponin, Sigma); or received saponin or saline. Three doses were administered at two-week intervals. Four weeks after the third and last immunization, animals (*n* = 4, per group) were euthanized to analyze the immune response elicited by vaccination. At the same time, the remaining animals were infected subcutaneously in the right hind footpad with 1 × 10^7^ stationary-phase promastigotes of *L. infantum*. Ten weeks after infection, animals were euthanized and the spleen, liver, bone marrow (BM), and lymph nodes draining the paws (dLN) of the animals were collected to evaluate the immune response and parasite burden. Vaccination experiments were repeated twice and presented similar results.

### Estimation of parasite burden

The liver, spleen, BM, and dLN were collected for parasite quantification, following a limiting-dilution technique, as previously described [19].

### Cytokine production before and after *L. infantum* infection

Splenocyte cultures and cytokine assays were performed after the last immunization and before infection, as well as at the 10th week after challenge. For this, single-cell suspensions were plated in duplicate in 24-well plates (Nunc), at 1 × 10^6^ cells per mL. Cells were incubated in RPMI 1640 medium (negative control), which was supplemented with 10 % FBS, 20 mM L-glutamine, 200 U/mL penicillin, and 100 μg/mL streptomycin, at pH 7.4; or separately stimulated with rLiHyV, Peptide1, Peptide2 or *L. infantum* SLA (20, 25, 25, or 25 μg mL^−1^, respectively); for 48 h at 37 °C in 5 % CO_2_. IFN-γ, IL-4, IL-10, IL-12, and GM-CSF levels were assessed in the supernatants by a sandwich ELISA provided in commercial kits (BD OptEIA TM set mouse IFN-γ, IL-4, IL-10, IL-12, and GM-CSF; all obtained from Pharmingen, San Diego, CA, USA); following manufacturer’s instructions. In order to block IL-12, CD4^+^, and CD8^+^ mediated T cell cytokine release, spleen cells of mice vaccinated with rLiHyV/saponin and challenged with *L. infantum* were *in vitro* stimulated with SLA (25 μg mL^−1^), and incubated in the presence of 5 μg mL^−1^ of monoclonal antibodies (mAb) against mouse IL-12 (C17.8), CD4 (GK 1.5), or CD8 (53–6.7). Appropriate isotype-matched controls – rat IgG2a (R35-95) and rat IgG2b (95–1) – were employed in the assays. Antibodies (no azide/low endotoxin^™^) were purchased from BD (Pharmingen).

### Evaluation of the humoral response

The antibody production was evaluated after the third and last immunization and before infection, as well as at the 10th week after challenge. The parasite-specific IgG1 and IgG2a isotype antibody levels were measured by an ELISA technique, as previously described [19].

### Statistical analysis

The results were entered into Microsoft Excel (version 10.0) spreadsheets, and analyzed using GraphPad Prism^™^ (version 6.0 for Windows). The mean optical density (O.D.) values were calculated by subtracting the mean blank O.D. from the mean value for each sample by using specific data obtained in the ELISA assays. The lower limits of positivity (cut-off) for rLiHyV and A2 proteins, as well as to SLA *L. infantum*, were established for optimal sensitivity and specificity using the Receiver Operator Curve (ROC curves). The accuracy was evaluated according to the area under the curve (AUC) relative to the ROC curve, with a 95 % confidence interval (CI95%). The ROC curves were plotted with the values from serum samples from dogs presenting CVL, when compared to those from the control groups (*T. cruzi*-, *E. canis*- or *B. canis*-infected, Leish-Tec®-immunized animals, and non-infected dogs), according to a sick/non-sick rating method. Statistical analysis of the data from vaccinated and/or infected animals was performed by one-way analysis of variance (ANOVA), following Bonferroni’s post-test for multiple comparisons between the groups. Results are shown as dotted or bars graphs, where mean ± standard deviation (SD) of each experimental group is shown. Differences were considered significant when *P* < 0.05.

## Results and discussion

### Serodiagnosis of CVL using the rLiHyV protein

Serological tests are currently recommended for the laboratorial diagnosis of CVL. Some of them, such as IFAT and ELISA, can present low sensitivity to detect cases from animals presenting low levels of antileishmanial antibodies, also their specificity can be hampered due tocross-reaction with antibodies in sera of animals developing other pathologies, such as Chagas’ disease, ehrlichiose, and babesiosis [35, 36]. In addition, in Brazil, there are two commercial vaccines against CVL, Leishmune® [37] and Leish-Tec® [15] and, although protective, they can induce seroconversion in the immunized animals, causing them to be classified as false-positive in the serological assays performed [9, 38].

**Fig. 1:**
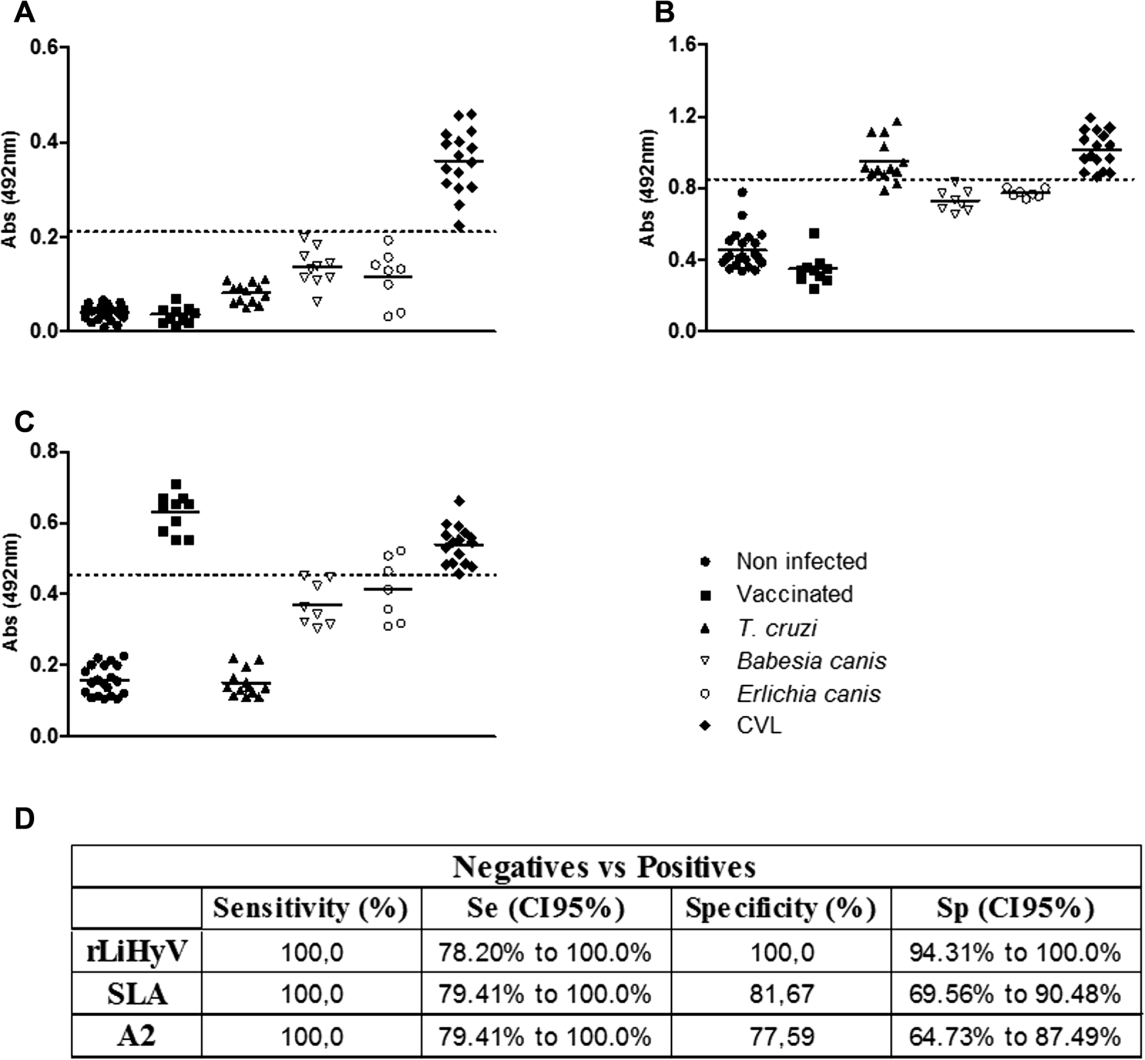
Diagnostic performance of LiHyV protein. ELISA assays were performed using sera samples of dogs with visceral leishmaniasis (*n* = 16), non-infected dogs living in an endemic area of leishmaniasis (*n* = 20), Leish-Tec®-vaccinated animals (*n* = 10) and animals experimentally infected by *Trypanosoma cruzi* (*n* = 13), *Ehrlichia canis* (*n* = 7) or *Babesia canis* (*n* = 7). The rLiHyV (**a**), SLA *L. infantum* (**b**), and rA2 (**c**) were used as antigens. The optical density (O.D.) values of each individual serum are shown. The cut-off values (*dotted line*) for negative and positive sample discrimination were calculated using the mean ± three times the standard deviation of all negative samples. The sensitivity and specificity values of the antigens were calculated and are also showed (**d**)

In the present study, a non-described *Leishmania*-specific protein was fused as a recombinant protein to an N-terminal 6x histidine-tag, and expressed in *E. coli*. The rLiHyV protein was purified and evaluated for the serodiagnosis of CVL. In the results, it was possible to verify that rLiHyV was recognized by all sera from VL dogs. In contrast, antibodies from *T. cruzi*-, *E. canis*- or *B. canis*-infected dogs, from Leish-Tec®-vaccinated animals or those of non-infected dogs; did not react with the recombinant protein (Fig. 1a). The SLA *L. infantum* and rA2 protein were used as comparative antigens. Using SLA, although all VL dogs’ sera had been recognized, a significant cross-reactivity was observed when sera from *T. cruzi-*infected animals were evaluated (Fig. 1b). Using the A2 protein as a diagnosis marker [39–41], a cross-reaction was also observed when sera of dogs experimentally infected with *E. canis* or *B. canis* were evaluated (Fig. 1c). To determine the diagnostic performance of the rLiHyV protein, ROC curves were constructed, and it was possible to observe that this antigen presented sensitivity and specificity values of 100 %; whereas using SLA, these values were of 100 and 81.67 %, respectively; and with the A2 protein, they were of 100 and 77.59 %, respectively (Fig. 1d). The number and variety of sera used in the present study could be considered a limiting factor. In this context, data here presented should be taken as a proof-of-concept of the efficacy of the proposed antigen to be employed in the serodiagnosis of CVL, and may well serve as a reference for further assays. On the other hand, we believe that, after an adequate validation, the rLiHyV protein may be promptly applied for a sensitive and specific serodiagnosis of CVL.

### Immunogenicity of LiHyV protein in BALB/c mice

**Fig. 2:**
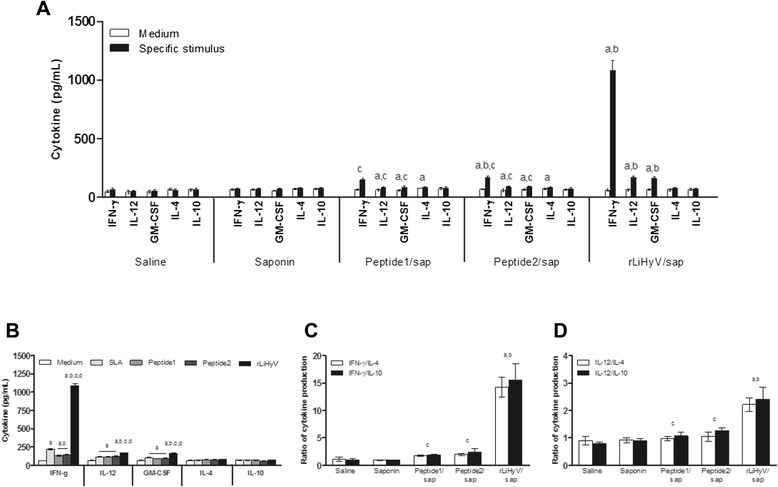
Immune response induced by immunization with rLiHyV protein plus saponin. Single cells suspensions were obtained from the spleen of mice, four weeks after the last immunization and before infection. Cells were unstimulated (*negative control*), or separately stimulated with rLiHyV (rLiHyV/saponin group, 20 μg/mL), Peptide1 (Peptide1/saponin group, 20 μg/mL) or Peptide2 (Peptide2/saponin group, 25 μg/mL). Saline and saponin groups were stimulated with SLA (25 μg/mL). IFN-γ, IL-12, GM-CSF, IL-4, and IL-10 levels were measured by a capture ELISA (**a**). Cytokine levels of spleen cells derived from animals immunized with rLiHyV/saponin and stimulated with rLiHyV, Peptide1, Peptide2 or SLA (**b**). Statistically significant differences (*P* < 0.001) were observed between negative control (*medium*), SLA, Peptide1 and Peptide2 (*a, b, c,* and *d,* respectively) and in ratios of IFN-γ/IL-10 and IFN-γ/IL-4 (**c**), as well as between IL-12/IL-10 and IL-12/IL-4 (**d**). Bars represent the mean ± standard deviation of the groups. Statistically significant differences (*P* < 0.05) in relation to the saline, saponin or rLiHyV/saponin groups were observed (*a, b* and *c* respectively)

In this study, the immunogenicity of rLiHyV protein, as well as two synthetic peptides (Peptide1 and Peptide2) covering two putative CD8^+^ T cell epitopes present in the primary structure of the protein was evaluated in BALB/c mice. Four weeks after the last vaccine dose and before infection, the immune response elicited by the vaccine was evaluated. Following the *in vitro* stimulation with the rLiHyV protein, spleen cells derived from mice vaccinated with rLiHyV/saponin produced significantly higher levels of IFN-γ, IL-12, and GM-CSF after rLiHyV stimulation, when compared to the negative control (background medium). Although the Peptide1/saponin and Peptide2/saponin groups had presented a high IFN-γ production after the specific stimulus, this production was comparatively lower in relation to that observed in the rLiHyV/saponin group. In addition, a low production of IL-4 and IL-10 was observed in all experimental groups (Fig. 2a).

**Fig. 3:**
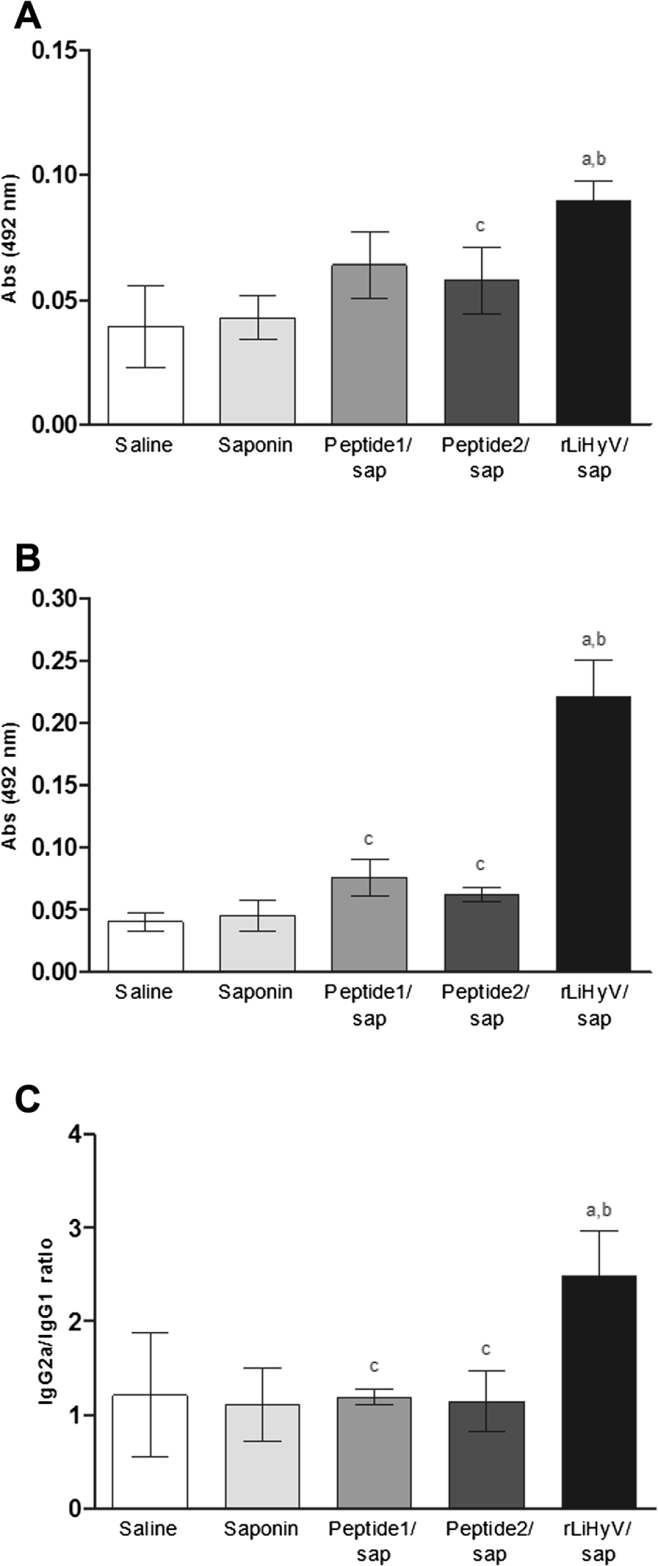
Humoral response induced by vaccination with rLiHyV protein plus saponin. Sera samples were collected from animals four weeks after the third and last immunization, and before infection. The parasite-specific IgG1 (**a**) and IgG2a (**b**) isotype levels were determined, and the bars represent the mean ± standard deviation of the groups. The ratios between IgG2a and IgG1 levels were also calculated (**c**). Statistically significant differences in relation to the saline, saponin or rLiHyV/saponin groups were observed (*a, b* and *c* letters, respectively; *P* < 0.05)

Using the different stimulations (rLiHyV, Peptide1, Peptide2 and SLA) in the spleen cells of mice of the rLiHyV/saponin group; it was observed that both Peptide1 and Peptide2 were able to induce a similar IFN-γ production, but that was significantly lower when compared to the levels found of this cytokine using the recombinant protein stimulus (Fig. 2b). Using the data collected from the specific stimulus performed in each experimental group, the ratios between IFN-γ/IL-4 and IFN-γ/IL-10 levels (Fig. 2c), as well as between IL-12/IL-4 and IL-12/IL-10 levels (Fig. 2d) were calculated, and the results showed that mice vaccinated with rLiHyV/saponin mounted a more pronounced Th1 response before infection, when compared to the other groups. In the evaluation of humoral response, mice vaccinated with rLiHyV/saponin produced higher levels of parasite-specific IgG1 (Fig. 3a) and IgG2a (Fig. 3b) isotype antibodies, when compared to the control groups. The ratio between the IgG2a and IgG1 levels was also calculated, and the results showed that mice vaccinated with rLiHyV/saponin presented a higher predominance of the IgG2a isotype in relation to its IgG1 levels (Fig. 3c). In this context, it could be concluded that the immunization with rLiHyV/saponin induced a higher *in vitro* IFN-γ, IL-12 and GM-CSF production, as well as low levels of IL-4, IL-10 and parasite-specific IgG1 antibodies in the vaccinated animals.

### Protective efficacy against *L. infantum* infection

**Fig. 4:**
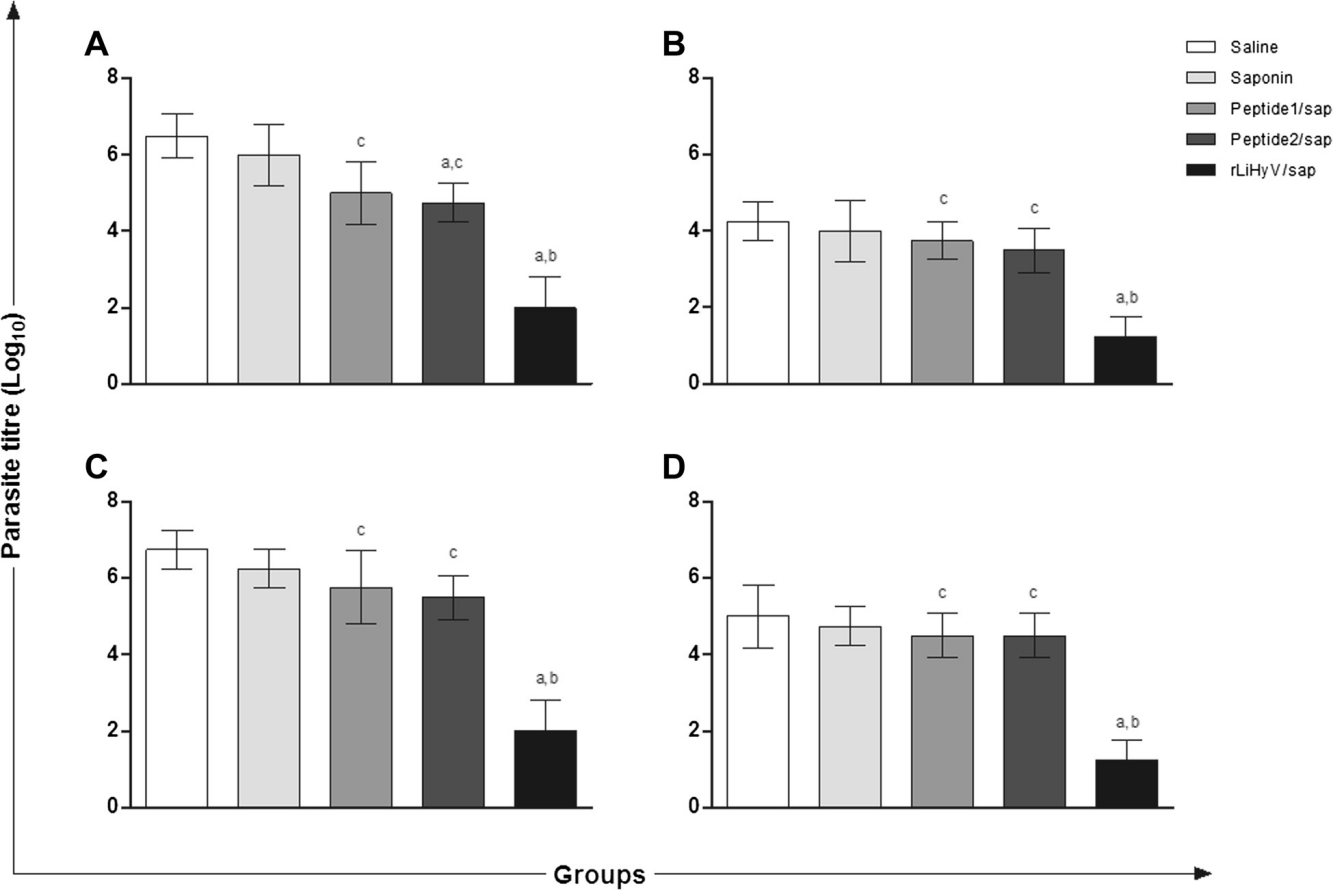
Protection of BALB/c mice vaccinated with rLiHyV protein plus saponin against *Leishmania infantum* infection*.* Mice were immunized and lately infected with *L. infantum.* The parasite burden in the liver (**a**), spleen (**b**), bone marrow (**c**), and lymph nodes draining the paws (**d**) was measured 10 weeks after challenge. Bars represent the mean ± standard deviation of the groups. Statistically significant differences in relation to the saline, saponin or rLiHyV/saponin groups were observed (*a, b* and *c* letters, respectively; *P* < 0.05)

To evaluate the protective efficacy of rLiHyV protein and its two peptides in BALB/c mice against *L. infantum* infection, the parasite load was estimated in the liver, spleen, BM, and dLN of the vaccinated and infected animals, 10 weeks after challenge. In the results, significant reductions in the parasite burden were observed in all organs of mice vaccinated with rLiHyV/saponin, when compared to the other groups (Fig. 4). In this context, mice vaccinated with rLiHyV/saponin presented reductions in the parasite load in the liver (3.4- and 3.2-log reductions, Fig. 4a), spleen (3.3- and 3.0-log reductions, Fig. 4b), BM (4.0- and 3.8-log reductions, Fig. 4c), and dLN (3.4- and 3.3-log reductions, Fig. 4d), when compared to the saline and saponin groups, respectively. Comparing the protective efficacy of the recombinant protein and its synthetic peptides, it was possible to verify that mice immunized with rLiHyV/saponin presented significant reductions in the parasite load in the liver (3.0- and 2.8-log reductions, Fig. 4a), spleen (2.5- and 2.4-log reductions, Fig. 4b), BM (3.6- and 3.6-log reductions, Fig. 4c), and dLN (2.9- and 2.8-log reductions, Fig. 4d), when compared to the Peptide1/saponin and Peptide2/saponin groups, respectively. These data were demonstrating that the inoculation of the recombinant protein was able to induce better protection than the peptide-derived vaccines.

With the advancement in recombinant DNA technology, *Leishmania spp*. proteins, either species or stage-specific of the parasites, had been evaluated as vaccine candidates against leishmaniasis. The major advantages associated to the use of recombinant proteins have been based in terms of standardization, purity and yield of production [24]. On the other hand, although whole proteins had been well-studied [15, 18, 21, 42], the protective immunity is known to be triggered by their CD4^+^ and/or CD8^+^ T cells epitopes [43–45]. More recent bioinformatics approaches utilize a number of algorithms for predicting epitopes, HLA-binding, transporter of antigen processing affinity, and proteasome cleavage to explore the use of these peptides with the higher probability to be immunogenic, and inducing protection against intracellular pathogens [46, 47]. In the present study, a comparison of the protective efficacy between the recombinant LiHyV protein and two of its putative CD8^+^ T cells-specific epitopes, Peptide1 and Peptide2, was performed. The choice to study CD8^+^ T cells epitopes was based on the fact that rLiHyV protein is also a protein expressed in the amastigote stage of *L. infantum*. In the results, it was obtained that the peptide-based vaccine was also able to reduce the parasite burden in the inoculated animals, when compared to the saponin and saline inoculated mice groups. These data may indicate the possibility of designing peptide-based vaccines. However, the protective characteristic of these vaccines should be improved, such as increasing the number of doses, the amount of peptide in each dose, and even using some carrier molecules [48–50]*.* On the other hand, employing combined vaccines containing different peptides could optimize the protective efficacy, when compared to the use of individual peptides [14, 24, 25, 51].

### Immunological parameters related with protection against infection

**Fig. 5:**
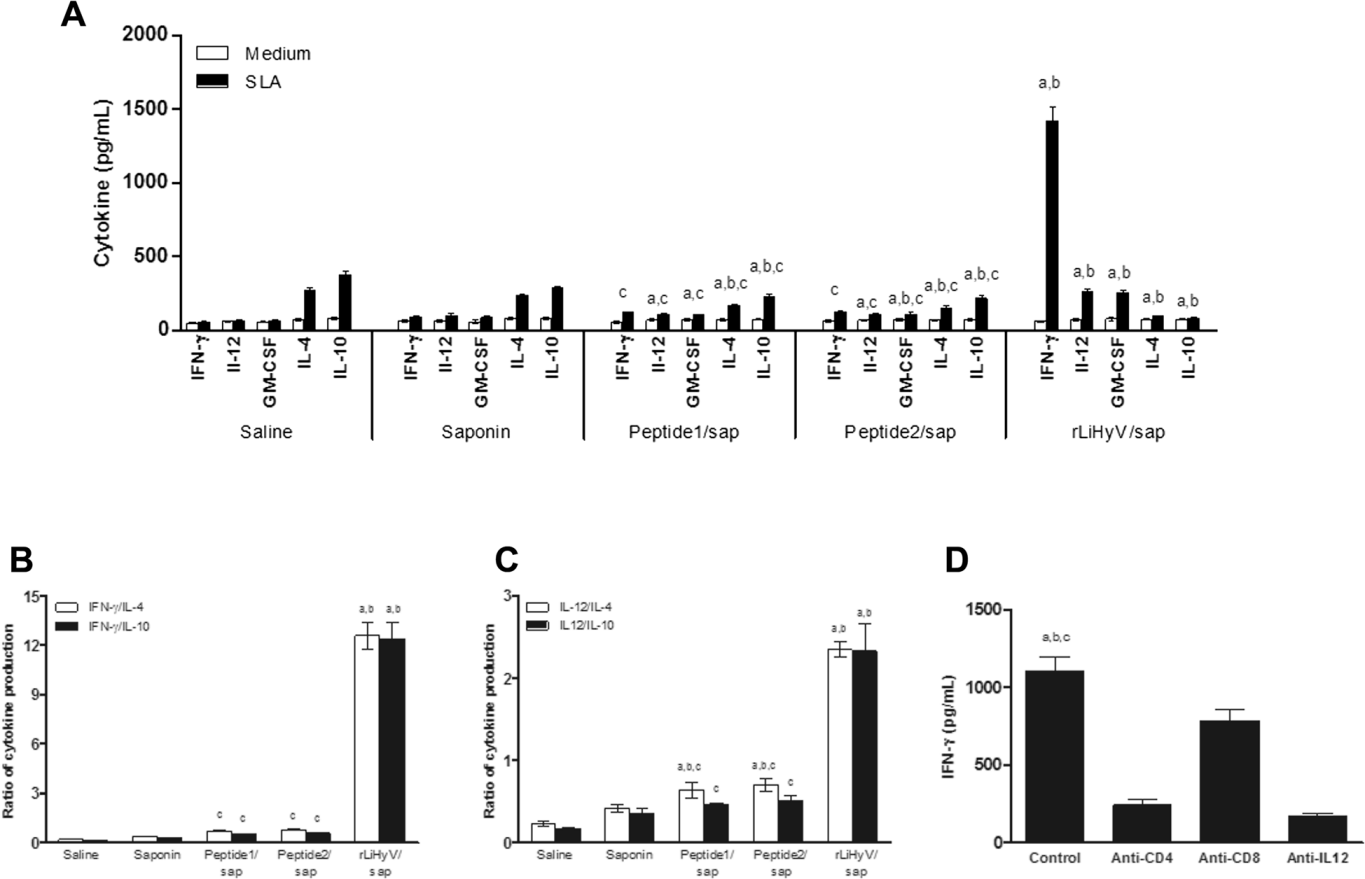
Analysis of cellular response and involvement of CD4^+^ and CD8^+^ T cells in the IFN-γ production after *L. infantum* infection. Spleen cells of mice were obtained 10 weeks after challenge, and were *in vitro* stimulated with SLA (25 μg per mL), for 48 h at 37 °C in 5 % CO_2_. IFN-γ, IL-12, GM-CSF, IL-4 and IL-10 levels were measured by a capture ELISA (**a**). The ratios between IFN-γ/IL-10 and IFN-γ/IL-4 levels (**b**), as well as between IL-12/IL-10 and IL-12/IL-4 levels (**c**) were calculated and also shown. Statistically significant differences in relation to the saline, saponin or rLiHyV/saponin groups were observed (*a, b* and *c* letters, respectively; *P* < 0.05). The involvement of CD4^+^ and CD8^+^ T cells in the IFN-γ production in the rLiHyV/saponin group was evaluated (**d**). Statistically significant differences in relation to the use of anti-IL-12, anti-CD4 or anti-CD8 monoclonal antibodies were observed (*a, b* and *c* letters, respectively; *P* < 0.05). Bars represent the mean ± standard deviation of the groups

The production of cytokines in the culture supernatants of spleen cells was also evaluated 10 weeks after infection. In the results, spleen cells of mice vaccinated with rLiHyV/saponin produced higher levels of parasite-specific IFN-γ, IL-12 and GM-CSF cytokines, than those secreted by spleen cells of the other groups (Fig. 5a). In contrast, in mice vaccinated with the rLiHyV/saponin, the SLA-driven production of IL-4 and IL-10 was lower when compared to animals that received saline or saponin (Fig. 5a). In addition, the ratios between IFN-γ/IL-4 and IFN-γ/IL-10 levels (Fig. 5b), as well as between IL-12/IL-4 and IL-12/IL-10 (Fig. 5c) levels demonstrated that mice vaccinated with rLiHyV/saponin mounted a more pronounced parasite-derived Th1 response after infection. The contribution of CD4^+^ and CD8^+^ T cells and the dependence of IL-12 production in the SLA-specific IFN-γ production of mice immunized with rLiHyV/saponin after infection was evaluated (Fig. 5d). The addition of anti-CD4 antibodies to the cultures was able to significantly decrease the parasite-specific IFN-γ production. A slight reduction was also observed when the anti-CD8 monoclonal antibody was used. These results indicate that the main production of IFN-γ is due to the induction of a parasite-specific CD4^+^ T cells response. These cells have proved to be an important source of IFN-γ in mice vaccinated with recombinant proteins. It is expected that these products are taken by phagocytic cells, processed and presented associated with MHC class II molecules, inducing a predominant CD4^+^ T cell response [50]. These results do not exclude the possibility of the existence of CD8^+^ epitopes in this protein, as expected by the protection observed in mice vaccinated with Peptide1 and Peptide2. For instance, it is possible to suggest that using the LiHyV sequence as a DNA vaccine, both CD4^+^ and CD8^+^ T cells responses could be generating, improving the vaccine protection based on a similar contribution of both T cells subtypes [52].

Spleen cells derived from BALB/c mice vaccinated with rLiHyV/saponin, when compared to the other groups, produced higher levels of SLA-specific GM-CSF, a cytokine related to macrophage activation and resistance against *L. major* [53], *L. donovani* [54], and *L. infantum* [16, 19, 21]. In addition, this cytokine has also played an important role in the activation, maturation, and function of dendritic cells [55]. The present study also showed that immunization using rLiHyV/saponin induced a low production of IL-4 and IL-10 after infection. Very low levels of parasite-specific IL-10 were detected after stimulation of spleen cells derived from vaccinated and infected mice, although spleen cells obtained from saline and saponin groups had showed a significantly higher production of these cytokines. Indeed, the control of parasite-mediated IL-10 response in mice may be critical for protection against murine VL, since this cytokine is considered to be the most important factor for inhibiting the disease progression in IL-10 deficient mice [56, 57], as well as in animals treated with an anti-IL-10 receptor antibody [58].

**Fig. 6:**
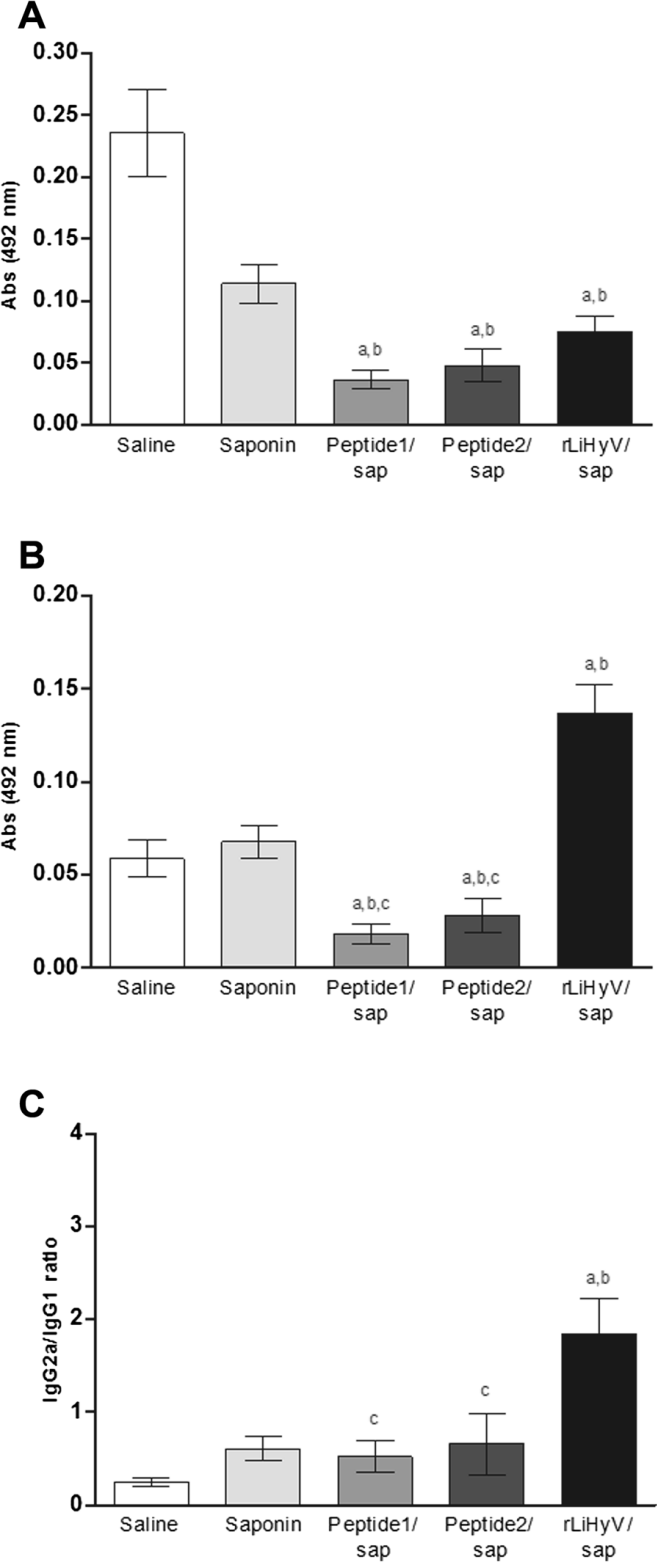
Analysis of humoral response after *L. infantum* infection. Sera samples were collected of animals, 10 weeks after infection, when the parasite-specific IgG1 (**a**) and IgG2a (**b**) isotype levels were evaluated. The ratios between the IgG2a and IgG1 levels were calculated, and the bars represent the mean ± standard deviation of the groups (**c**). Statistically significant differences in relation to the saline, saponin or rLiHyV/saponin groups were observed (*a, b* and *c* letters, respectively; *P* < 0.05)

In BALB/c mice, IL-4-dependent production of IgG1 antibodies is associated with disease progression caused by *L. amazonensis* [32, 43, 59] and *L. infantum* [19, 21, 60]. For instance, BALB/c mice vaccinated with the recombinant A2 protein [15, 32] or *Leishmania* ribosomal proteins [16] develop low levels of parasite-specific IgG1 antibodies, and this fact could contribute to protect the animals against *Leishmania spp*. infection. In the present study, mice that received saline or saponin presented higher parasite-specific IgG1 antibody levels (Fig. 6a), when compared to the other groups. On the other hand, animals vaccinated with rLiHyV/saponin showed higher levels of specific IgG2a antibodies (Fig. 6b), which possibly contributed to the protective response. These animals also presented a higher ratio between the IgG2a and IgG1 levels (Fig. 6c), demonstrating that the recombinant protein was able to mount a more pronounced Th1 response in the vaccinated animals, which was maintained after *L. infantum* infection.

## Conclusions

In the present study, a non-described *Leishmania*-specific protein, LiHyV, which was found to be expressed in both promastigote and amastigote stages of *L. infantum*, was successfully evaluated as a vaccine candidate against VL. Also, the recombinant protein demonstrated to be an effective marker for the serodiagnosis of CVL, and may well be employed in future studies aiming both immunological applications on leishmaniasis.
